# Supplementary material for: Inverse‐Intensity‐Weighted Generalized Estimating Equations With Irregularly Measured Longitudinal Data and Informative Dropout
Source: Stat Med. 2026 Jul 21;45(18-19):e70674. doi: 10.1002/sim.70674 (PMC13387368; doi:10.1002/sim.70674)
Supplement: Supplementary file 1 — Figure A1 Bias for AUC in Scenario 1 with η1=(0.5,1,1.5)⊤ and varying η0 to achieve different proportions of informative dropout. All three methods are compared, with our method in blue. 99.9th percentile‐trimmed weights are shown along with untrimmed weights for each case. Figure A2 Bias for AUC in Scenario 2 with η1=(−0.5,−1,−1.5)⊤ and varying η0 to achieve different proportions of informative dropout. All three methods are compared, with our method in blue. 99.9th percentile‐trimmed weights are shown along with untrimmed weights. Figure A3 Empirical standard errors for AUC in Scenario 1, with η1=(0.5,1,1.5)⊤ and varying η0 to achieve different proportions of informative dropout. All three methods are compared, with our method in blue. 99.9th percentile‐trimmed weights are shown along with untrimmed weights. Figure A4 Empirical standard errors for AUC in Scenario 2, with η1=(−0.5,−1,−1.5)⊤ and varying η0 to achieve different proportions of informative dropout. All three methods are compared, with our method in blue. 99.9th percentile‐trimmed weights are shown along with untrimmed weights. Figure A5 Coverage probabilities (CP) for AUC in Scenario 1 using naive standard errors, with η1=(0.5,1,1.5)⊤ and varying η0 to achieve different proportions of informative dropout. All three methods are compared, with our method in blue. 99.9th percentile‐trimmed weights are shown along with untrimmed weights. The dashed line represents 95% CP. Figure A6 Coverage probabilities (CP) for AUC in Scenario 2 using naive standard errors, with η1=(−0.5,−1,−1.5)⊤ and varying η0 to achieve different proportions of informative dropout. All three methods are compared, with our method in blue. 99.9th percentile‐trimmed weights are shown along with untrimmed weights. The dashed line represents 95% CP. Figure A7 Ratio of naive to empirical standard errors for AUC in Scenario 1, with η1=(0.5,1,1.5)⊤ and varying η0 to achieve different proportions of informative dropout. All three methods are c [file SIM-45-0-s001.pdf]

How to cite this article: Stefan G and Pullenayegum E. Inverse-intensity weighted generalized estimating equations with irregularly measured longitudinal data and informative dropout *Stat Med.* 2026;00(00):1–18.

APPENDIX

A OTHER SIMULATION RESULTS

For Scenario 2, the outcome is generated from the random-intercept model

$$Y_i(t) = \beta_0 + \beta_1(1+t)^{-2} + \beta_2(1+t)^{-2}\log(1+t) + b_i + \varepsilon_i(t), \quad b_i \sim N(0, \sigma_\phi^2), \quad \varepsilon_i(t) \sim N(0, \sigma_\varepsilon^2).$$

As in Scenario 1, we set $\lambda_0 = 1, \sigma_\phi = 1, \sigma_\varepsilon = 2$. In contrast with Scenario 1, we set $\gamma_0 = 0.5, \boldsymbol{\beta}_0 = (3.3, 4, 10.5)^\top, \tau = 3.5, c = 3$. Instead of setting positive values for η_1, we consider -0.5, -1, and -1.5. We adjust η_0 accordingly to achieve four informative dropout proportions: 20%, 40%, 60%, and 80%.

The figures described in this paragraph depict various performance metrics for all three methods discussed in the paper, with weights untrimmed and trimmed at the 99.9th percentile, across all configurations of η_0 and η_1. Simulations were performed with 20,000 iterations for four sample sizes: 200, 500, 1000, and 2000. Figures A1 and A2 depict the bias when estimating area under the curve (AUC) for Scenarios 1 and 2, respectively. Figures A3 and A4 depict the empirical standard errors (SEs)—standard deviation of the estimated AUCs across simulations—for Scenarios 1 and 2, respectively. Figures A5 and A6 depict the coverage probabilities—proportion of 95% confidence intervals for the AUC across simulations containing the true AUC value—for Scenarios 1 and 2, respectively. Figures A7 and A8 depict the ratio of naive to empirical SEs when estimating AUC for Scenarios 1 and 2, respectively. Here, naive SEs are computed as the mean of the robust SEs obtained from the GEE models across simulations; these do not take into account uncertainty from estimating the weights.

Tables A1 and A2 summarize the previously described performance metrics for all methods, four levels of trimming, and three configurations of $\boldsymbol{\eta}$, further comparing with bootstrap standard errors and coverage probabilities. Due to time constraints related to the bootstrap, only 1,000 simulation iterations were performed. Since the objective was to assess performance of the various standard errors, a relatively small sample size of 200 was used. The bootstrap was performed within each simulation iteration with 100 bootstrap iterations and randomly sampling 200 individuals with replacement.

Figures A9 and A10 depict bias, coverage probabilities, and ratio of naive to empirical standard errors when estimating AUC for Scenarios 1 and 2, respectively, for all three methods, with weights untrimmed and trimmed at the 99.9th percentile, when the dropout model is misspecified; we define a time-varying slope term $\eta_1(t)$ for both scenarios but specify it as constant in the model. We vary the intercept term η_0 to achieve a wide range of dropout proportions. Simulations were performed with 20,000 iterations for four sample sizes: 200, 500, 1000, and 2000.

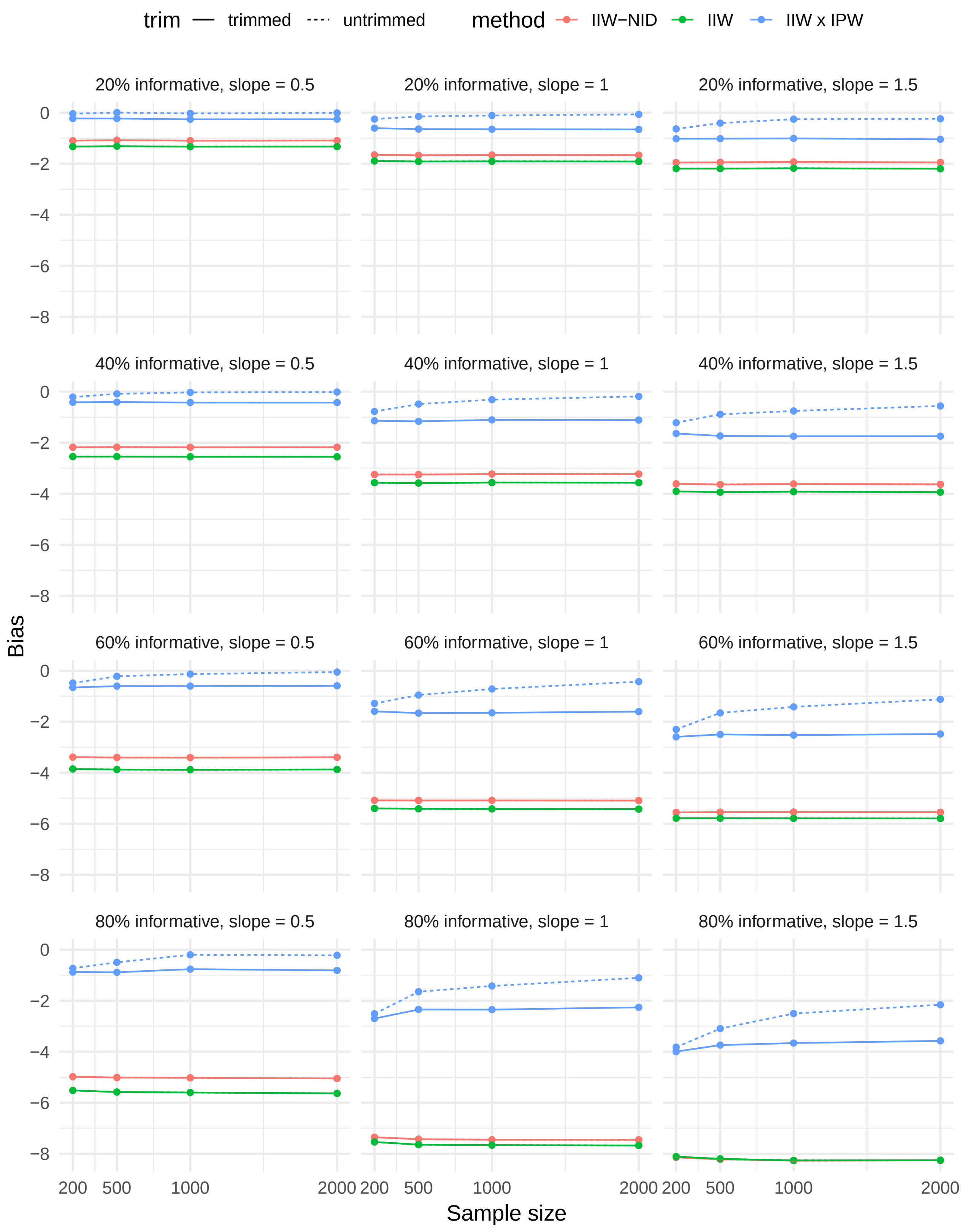

FIGURE A1 Bias for AUC in Scenario 1 with $\eta_1 = (0.5, 1, 1.5)^\top$ and varying η_0 to achieve different proportions of informative dropout. All three methods are compared, with our method in blue. 99.9th percentile-trimmed weights are shown along with untrimmed weights for each case.

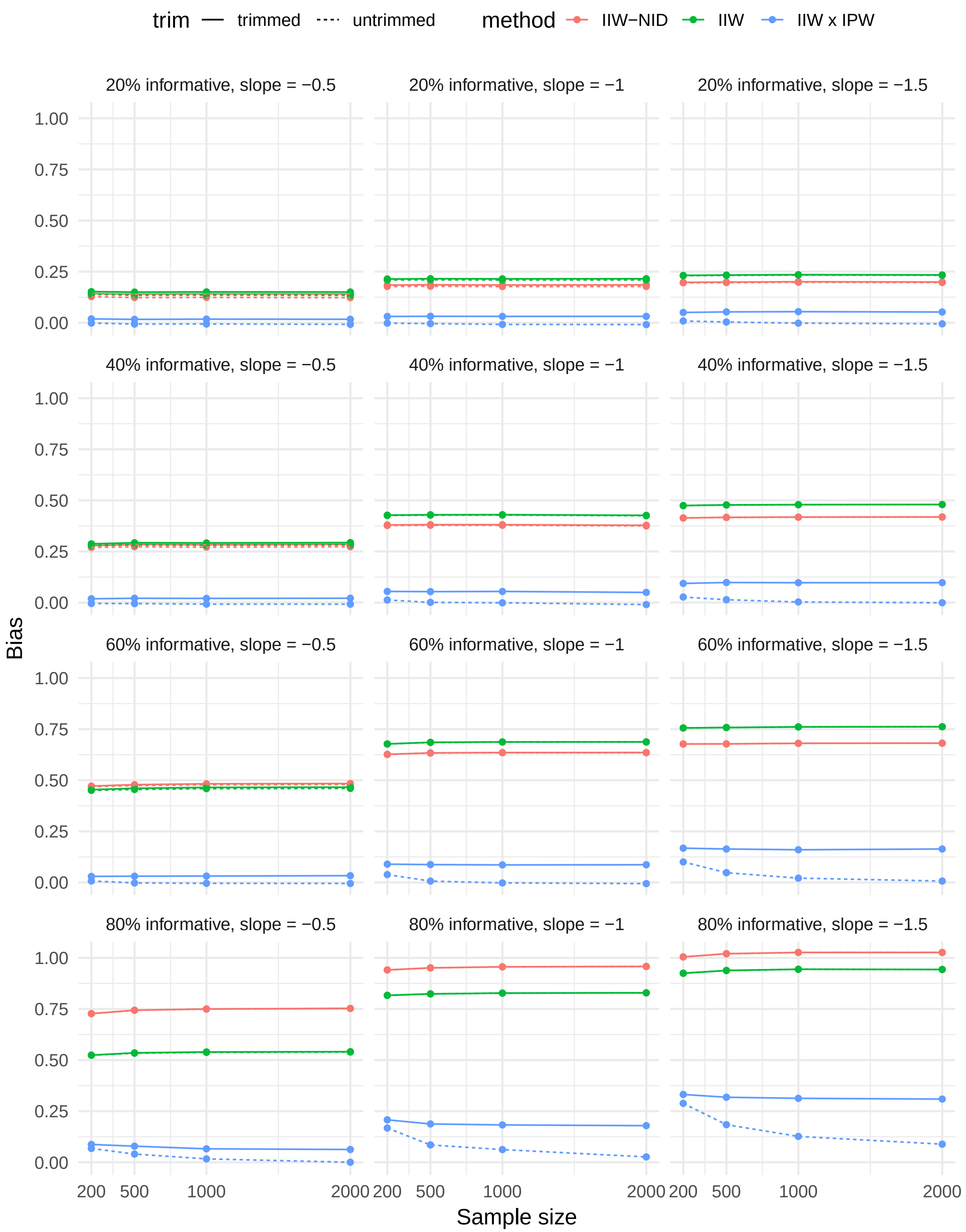

FIGURE A2 Bias for AUC in Scenario 2 with $\eta_1 = (-0.5, -1, -1.5)^\top$ and varying η_0 to achieve different proportions of informative dropout. All three methods are compared, with our method in blue. 99.9th percentile-trimmed weights are shown along with untrimmed weights.

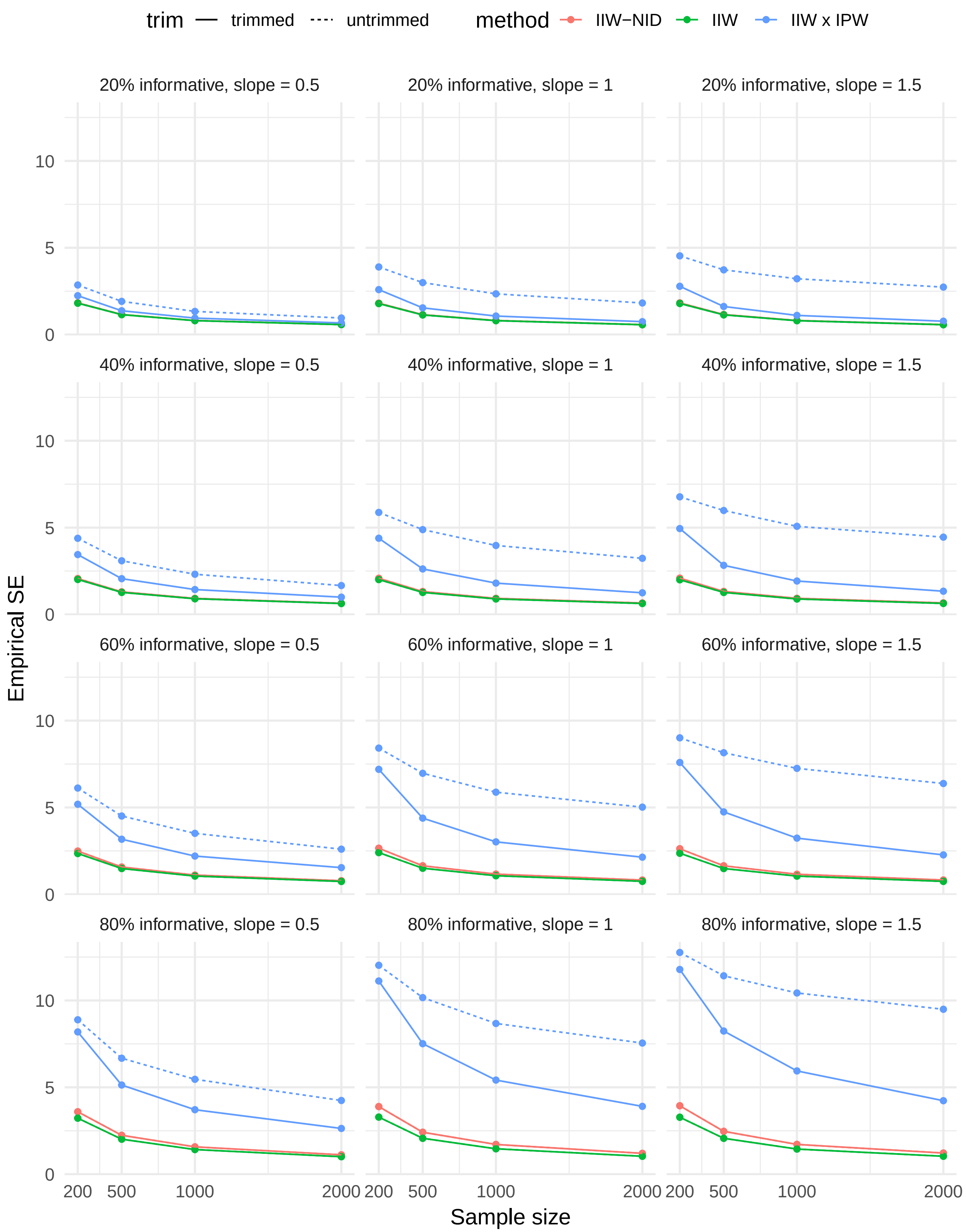

FIGURE A3 Empirical standard errors for AUC in Scenario 1, with $\eta_1 = (0.5, 1, 1.5)^\top$ and varying η_0 to achieve different proportions of informative dropout. All three methods are compared, with our method in blue. 99.9th percentile-trimmed weights are shown along with untrimmed weights.

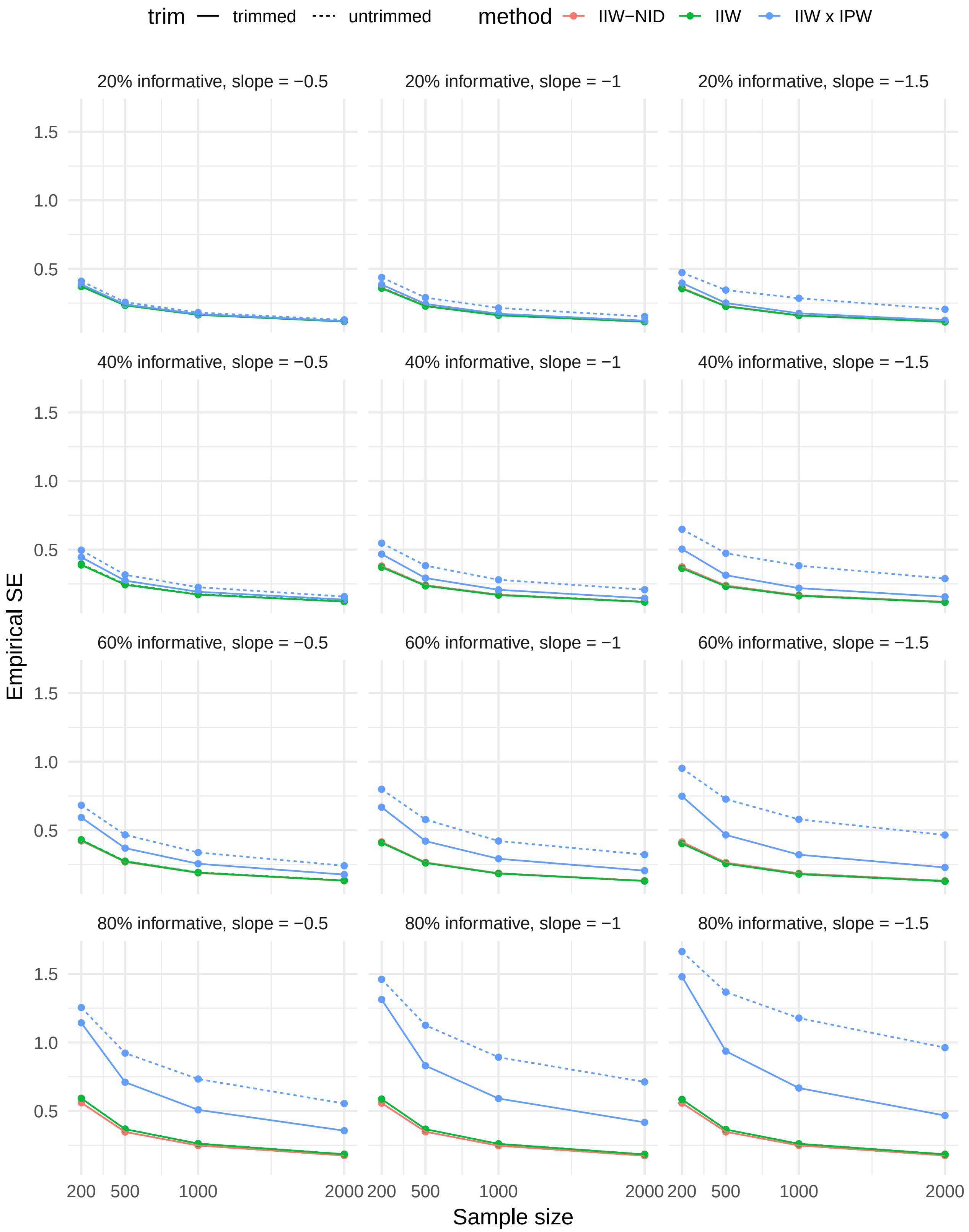

FIGURE A4 Empirical standard errors for AUC in Scenario 2, with $\eta_1 = (-0.5, -1, -1.5)^\top$ and varying η_0 to achieve different proportions of informative dropout. All three methods are compared, with our method in blue. 99.9th percentile-trimmed weights are shown along with untrimmed weights.

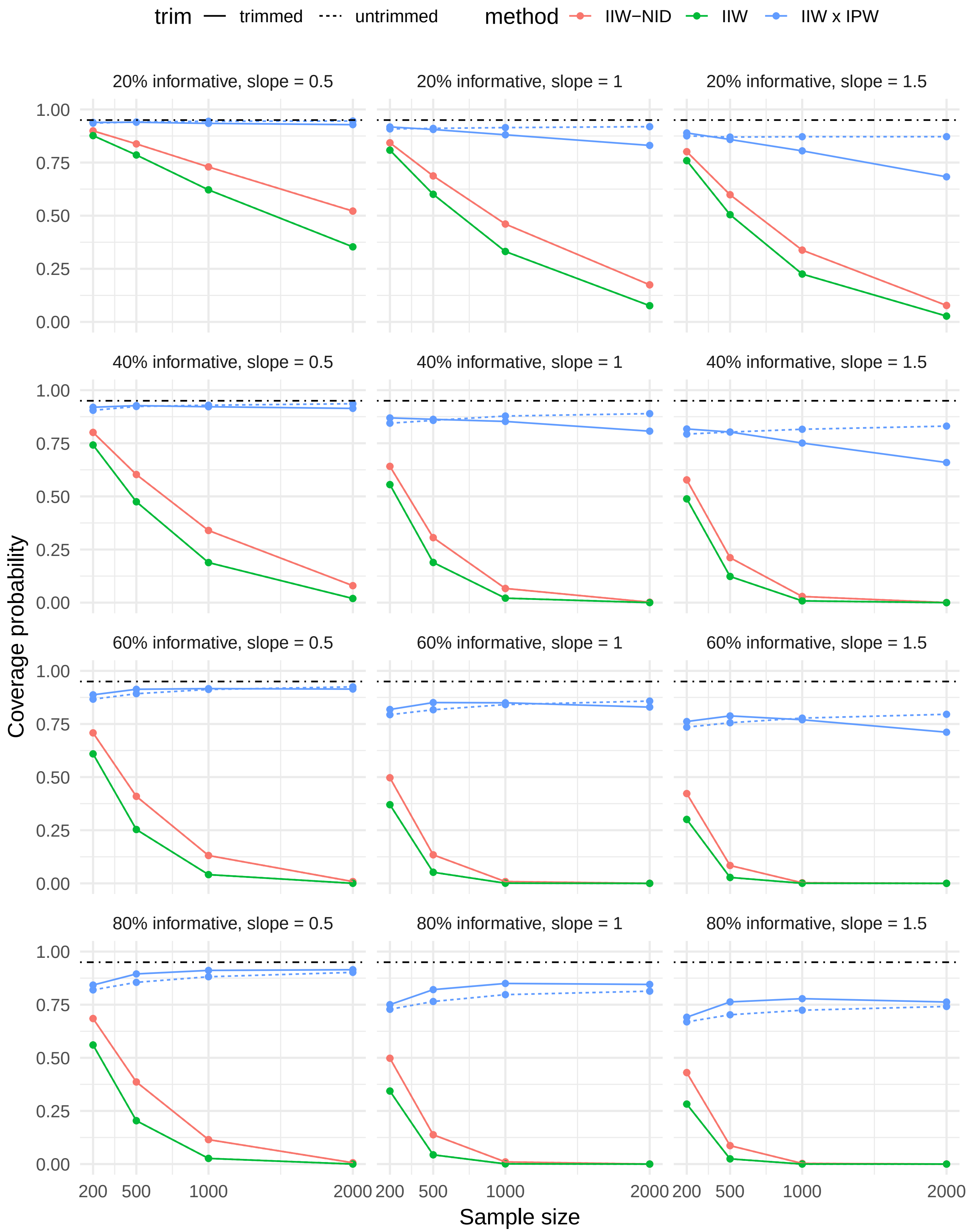

FIGURE A5 Coverage probabilities (CP) for AUC in Scenario 1 using naive standard errors, with $\eta_1 = (0.5, 1, 1.5)^\top$ and varying η_0 to achieve different proportions of informative dropout. All three methods are compared, with our method in blue. 99.9th percentile-trimmed weights are shown along with untrimmed weights. The dashed line represents 95% CP.

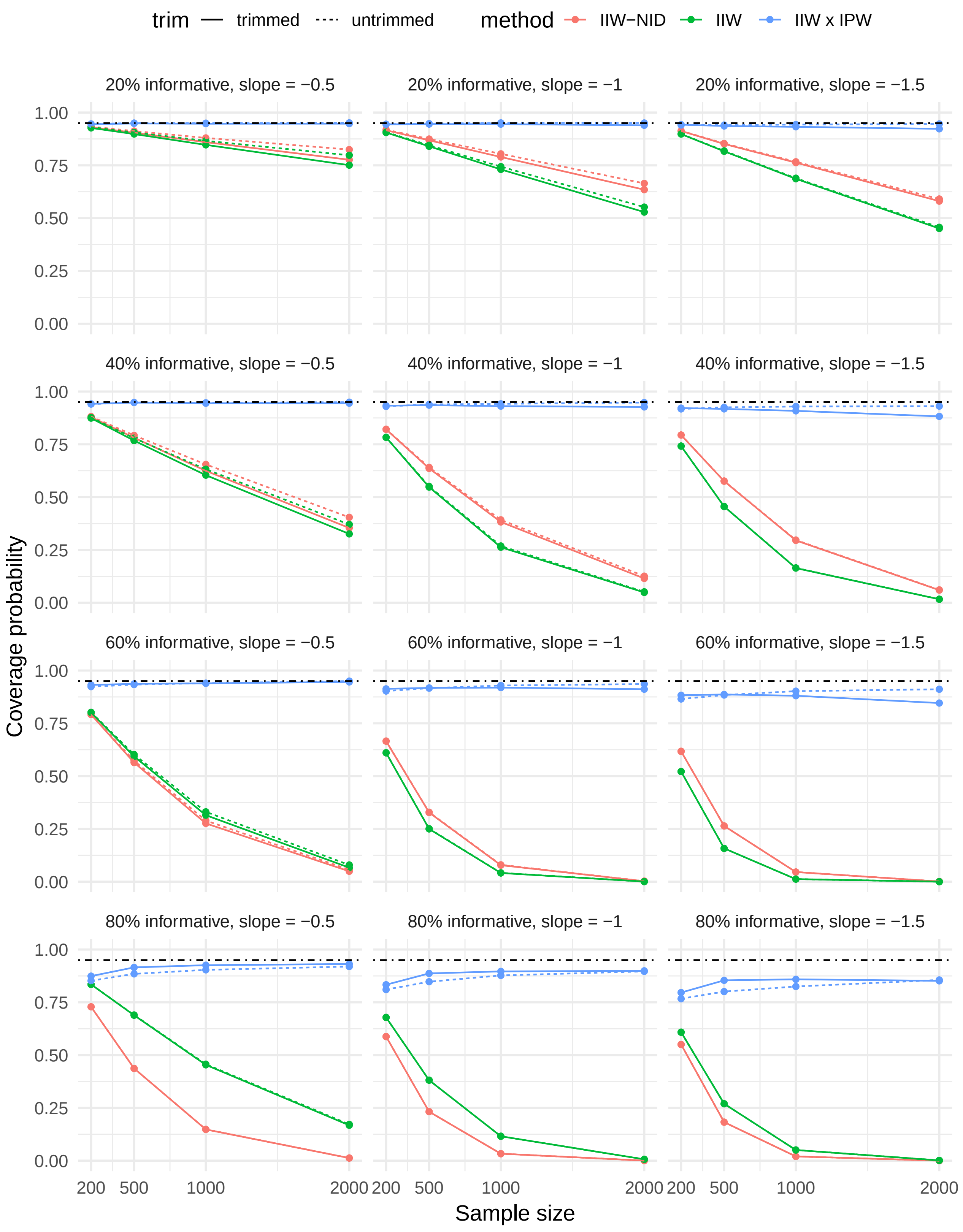

FIGURE A6 Coverage probabilities (CP) for AUC in Scenario 2 using naive standard errors, with $\eta_1 = (-0.5, -1, -1.5)^\top$ and varying η_0 to achieve different proportions of informative dropout. All three methods are compared, with our method in blue. 99.9th percentile-trimmed weights are shown along with untrimmed weights. The dashed line represents 95% CP.

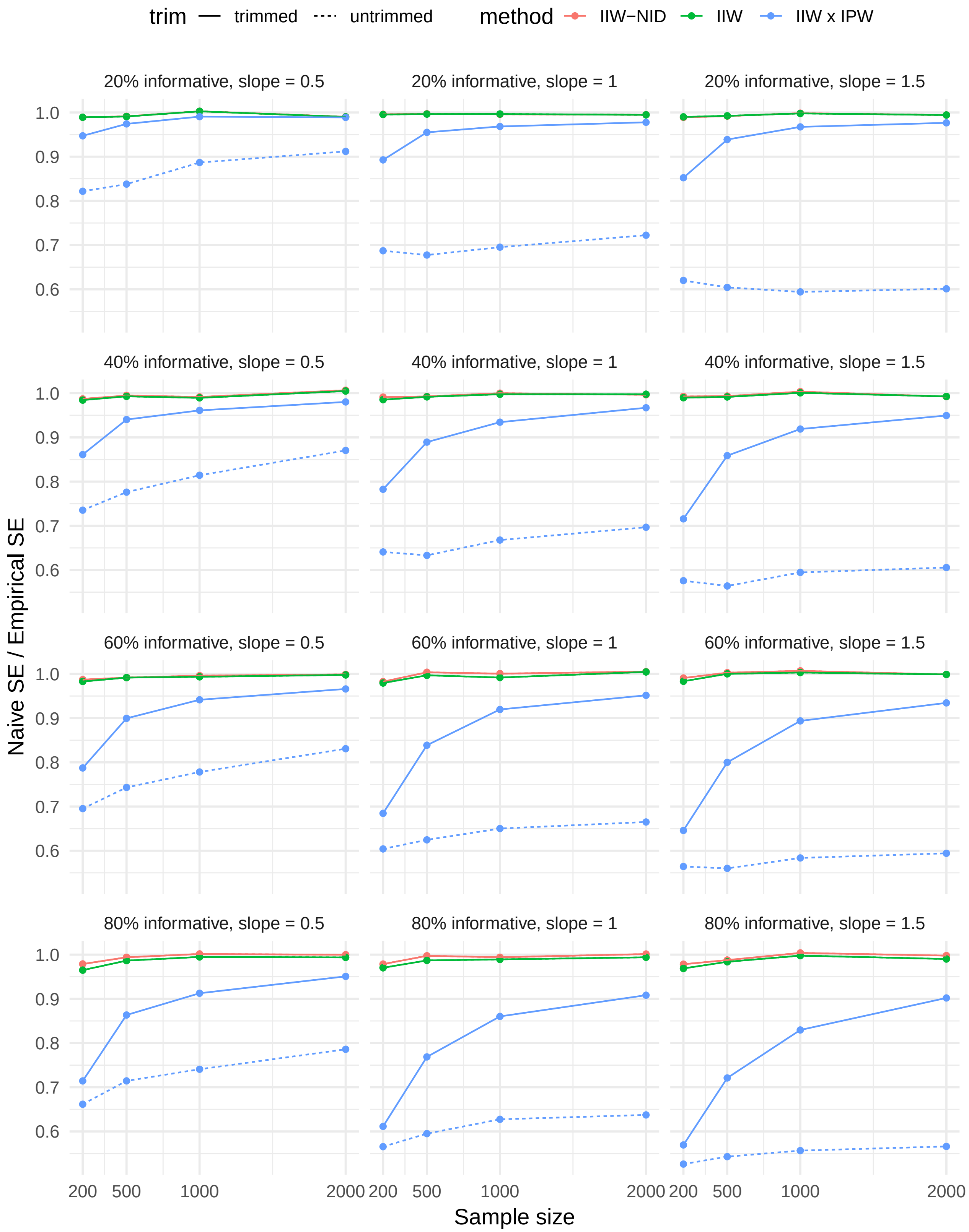

FIGURE A7 Ratio of naive to empirical standard errors for AUC in Scenario 1, with $\eta_1 = (0.5, 1, 1.5)^\top$ and varying η_0 to achieve different proportions of informative dropout. All three methods are compared, with our method in blue. 99.9th percentile-trimmed weights are shown along with untrimmed weights.

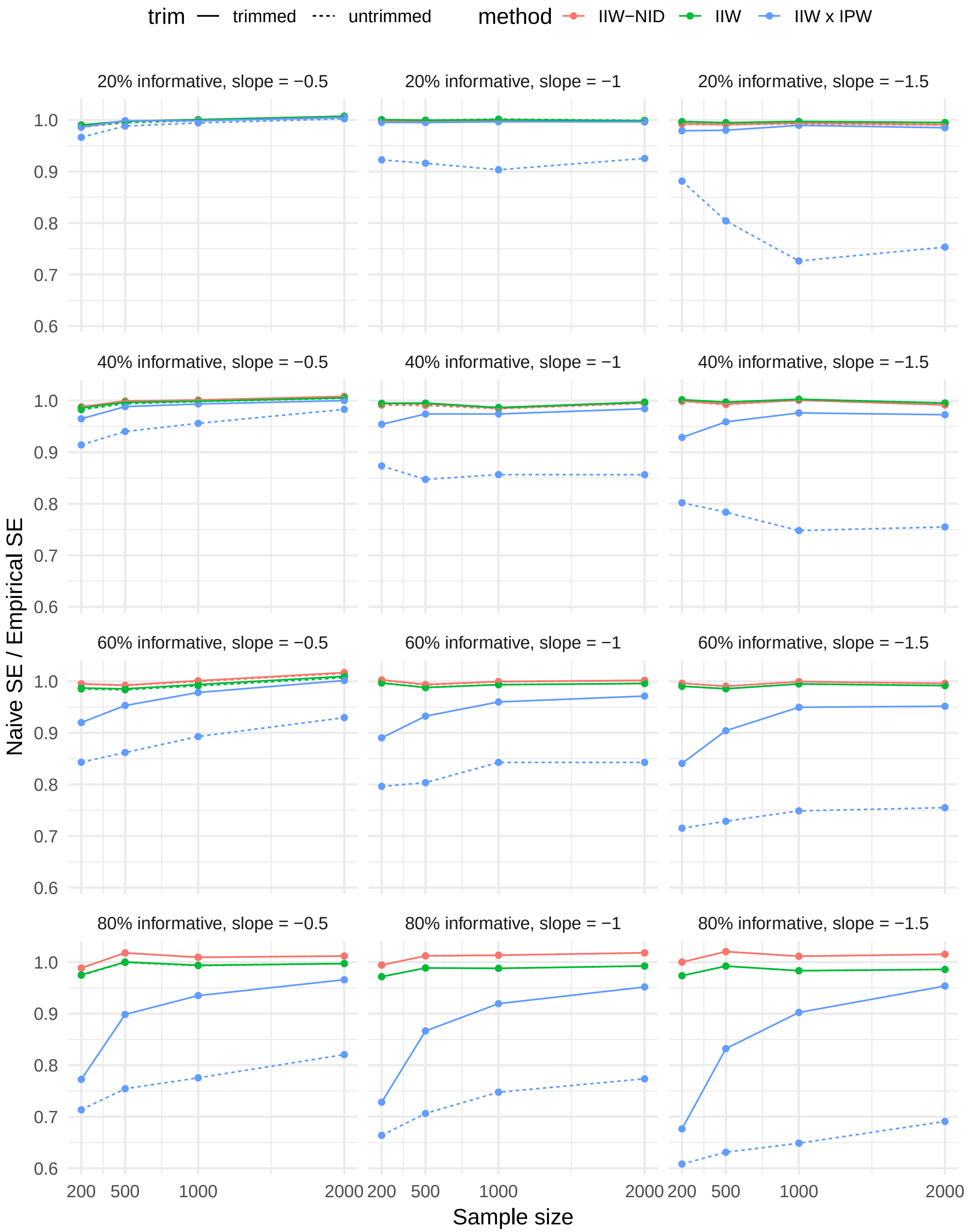

FIGURE A8 Ratio of naive to empirical standard errors for AUC in Scenario 2, with $\eta_1 = (-0.5, -1, -1.5)^\top$ and varying η_0 to achieve different proportions of informative dropout. All three methods are compared, with our method in blue. 99.9th percentile-trimmed weights are shown along with untrimmed weights.

% inf	Slope	Trim	Model	Bias	Emp SE	Naive SE	CP	Boot SE	Boot CP	Boot SD
20%	0.5	None	IIW-NID	-1.1	1.8	1.8	0.90	1.8	0.90	0.19
			IIW	-1.3	1.8	1.8	0.88	1.8	0.87	0.19
			IIW×IPW	-0.2	2.8	2.3	0.94	2.5	0.96	1.1
20%	0.5	99.9%	IIW-NID	-1.1	1.8	1.8	0.90	1.8	0.90	0.19
			IIW	-1.3	1.8	1.8	0.88	1.8	0.87	0.19
			IIW×IPW	-0.3	2.1	2.1	0.95	2.4	0.97	1.0
20%	0.5	99.5%	IIW-NID	-1.1	1.8	1.8	0.90	1.8	0.90	0.19
			IIW	-1.3	1.8	1.8	0.88	1.8	0.87	0.19
			IIW×IPW	-0.5	1.9	1.9	0.94	2.0	0.95	0.36
20%	0.5	99%	IIW-NID	-1.1	1.8	1.8	0.90	1.8	0.90	0.19
			IIW	-1.3	1.8	1.8	0.88	1.8	0.87	0.19
			IIW×IPW	-0.6	1.8	1.9	0.95	1.9	0.95	0.22
60%	1.0	None	IIW-NID	-5.0	2.6	2.6	0.51	2.6	0.52	0.35
			IIW	-5.3	2.4	2.4	0.37	2.4	0.37	0.31
			IIW×IPW	-1.3	8.4	5.0	0.78	5.6	0.84	3.0
60%	1.0	99.9%	IIW-NID	-5.0	2.6	2.6	0.51	2.6	0.52	0.35
			IIW	-5.3	2.4	2.4	0.37	2.4	0.37	0.31
			IIW×IPW	-1.6	7.1	4.9	0.81	5.4	0.87	2.8
60%	1.0	99.5%	IIW-NID	-5.0	2.6	2.6	0.51	2.6	0.52	0.35
			IIW	-5.3	2.4	2.4	0.36	2.4	0.37	0.31
			IIW×IPW	-2.6	4.4	3.9	0.83	4.6	0.88	1.7
60%	1.0	99%	IIW-NID	-5.0	2.6	2.6	0.51	2.6	0.52	0.35
			IIW	-5.3	2.4	2.4	0.37	2.4	0.37	0.31
			IIW×IPW	-3.0	3.6	3.4	0.81	3.7	0.84	1.0
80%	1.5	None	IIW-NID	-8.1	3.8	3.9	0.44	4.0	0.46	0.67
			IIW	-8.1	3.2	3.2	0.28	3.3	0.29	0.59
			IIW×IPW	-3.4	14	6.8	0.69	8.2	0.78	4.9
80%	1.5	99.9%	IIW-NID	-8.1	3.8	3.9	0.44	4.0	0.46	0.67
			IIW	-8.1	3.2	3.2	0.28	3.3	0.29	0.59
			IIW×IPW	-3.6	12	6.8	0.70	8.1	0.80	4.6
80%	1.5	99.5%	IIW-NID	-8.1	3.8	3.9	0.44	4.0	0.46	0.67
			IIW	-8.1	3.2	3.2	0.28	3.3	0.29	0.59
			IIW×IPW	-4.7	7.2	5.9	0.75	7.3	0.83	3.5
80%	1.5	99%	IIW-NID	-8.1	3.8	3.9	0.44	4.0	0.46	0.67
			IIW	-8.1	3.2	3.2	0.28	3.3	0.29	0.59
			IIW×IPW	-5.4	5.7	5.1	0.73	5.9	0.78	2.1

TABLE A1 AUC for Scenario 1 with $n_{\text{sim}} = 1000, n = 200, \gamma_0 = -0.336, \boldsymbol{\beta}_0 = (16.4, -3.1)^\top, \tau = 16, c = 2, \lambda_0 = 1, \sigma_\phi = 1, \sigma_\varepsilon = 2$. % inf is the percentage of subjects who were informatively censored and the slope η_1 controls "informativeness." Bias is presented along with the empirical standard errors. Further, we present the naive standard errors and their corresponding coverage probabilities (CP), as well as the bootstrap standard errors along with their corresponding CPs. The standard deviation of the bootstrap standard errors is shown in the rightmost column. All three methods are compared, with four levels of trimming.

% inf	Slope	Trim	Model	Bias	Emp SE	Naive SE	CP	Boot SE	Boot CP	Boot SD
20%	−0.5	None	IIW-NID	0.11	0.38	0.38	0.92	0.37	0.93	0.05
			IIW	0.12	0.38	0.37	0.92	0.37	0.93	0.05
			IIW×IPW	-0.02	0.41	0.40	0.95	0.40	0.94	0.08
20%	−0.5	99.9%	IIW-NID	0.12	0.38	0.37	0.92	0.38	0.93	0.05
			IIW	0.13	0.38	0.37	0.92	0.37	0.92	0.05
			IIW×IPW	0.00	0.39	0.38	0.95	0.40	0.95	0.08
20%	−0.5	99.5%	IIW-NID	0.13	0.37	0.37	0.92	0.37	0.92	0.04
			IIW	0.14	0.37	0.37	0.92	0.37	0.92	0.04
			IIW×IPW	0.02	0.38	0.37	0.94	0.38	0.94	0.04
20%	−0.5	99%	IIW-NID	0.13	0.37	0.37	0.92	0.37	0.92	0.04
			IIW	0.14	0.37	0.37	0.92	0.37	0.92	0.04
			IIW×IPW	0.04	0.38	0.37	0.95	0.37	0.94	0.04
60%	−1.0	None	IIW-NID	0.64	0.42	0.42	0.64	0.42	0.65	0.05
			IIW	0.69	0.42	0.41	0.59	0.41	0.60	0.05
			IIW×IPW	0.05	0.77	0.63	0.92	0.64	0.93	0.24
60%	−1.0	99.9%	IIW-NID	0.64	0.42	0.42	0.64	0.42	0.65	0.05
			IIW	0.69	0.42	0.41	0.59	0.41	0.60	0.05
			IIW×IPW	0.10	0.66	0.59	0.93	0.64	0.95	0.23
60%	−1.0	99.5%	IIW-NID	0.64	0.42	0.42	0.64	0.42	0.65	0.05
			IIW	0.69	0.42	0.41	0.59	0.41	0.60	0.05
			IIW×IPW	0.21	0.53	0.51	0.91	0.55	0.93	0.13
60%	−1.0	99%	IIW-NID	0.64	0.42	0.42	0.64	0.42	0.65	0.05
			IIW	0.69	0.42	0.41	0.59	0.41	0.60	0.05
			IIW×IPW	0.28	0.47	0.47	0.89	0.50	0.91	0.08
80%	−1.5	None	IIW-NID	1.0	0.55	0.56	0.54	0.55	0.53	0.07
			IIW	0.95	0.57	0.57	0.60	0.57	0.61	0.08
			IIW×IPW	0.29	1.6	1.0	0.79	1.1	0.85	0.55
80%	−1.5	99.9%	IIW-NID	1.0	0.55	0.56	0.54	0.55	0.53	0.07
			IIW	0.95	0.57	0.57	0.60	0.57	0.61	0.08
			IIW×IPW	0.33	1.4	1.0	0.81	1.1	0.87	0.52
80%	−1.5	99.5%	IIW-NID	1.0	0.55	0.56	0.54	0.55	0.53	0.07
			IIW	0.95	0.57	0.57	0.60	0.57	0.61	0.08
			IIW×IPW	0.52	0.94	0.85	0.84	1.0	0.88	0.36
80%	−1.5	99%	IIW-NID	1.0	0.55	0.56	0.54	0.55	0.53	0.07
			IIW	0.95	0.57	0.57	0.60	0.57	0.61	0.08
			IIW×IPW	0.62	0.76	0.74	0.82	0.83	0.87	0.22

TABLE A2 AUC for Scenario 2 with $n_{\text{sim}} = 1000, n = 200, \gamma_0 = 0.5, \beta_0 = (3.3, 4, 10.5)^\top, \tau = 3.5, c = 3, \lambda_0 = 1, \sigma_\phi = 1, \sigma_\varepsilon = 2$. % inf is the percentage of subjects who were informatively censored and the slope η_1 controls "informativeness." Bias is presented along with the empirical standard errors. Further, we present the naive standard errors and their corresponding coverage probabilities (CP), as well as the bootstrap standard errors along with their corresponding CPs. The standard deviation of the bootstrap standard errors is shown in the rightmost column. All three methods are compared, with four levels of trimming.

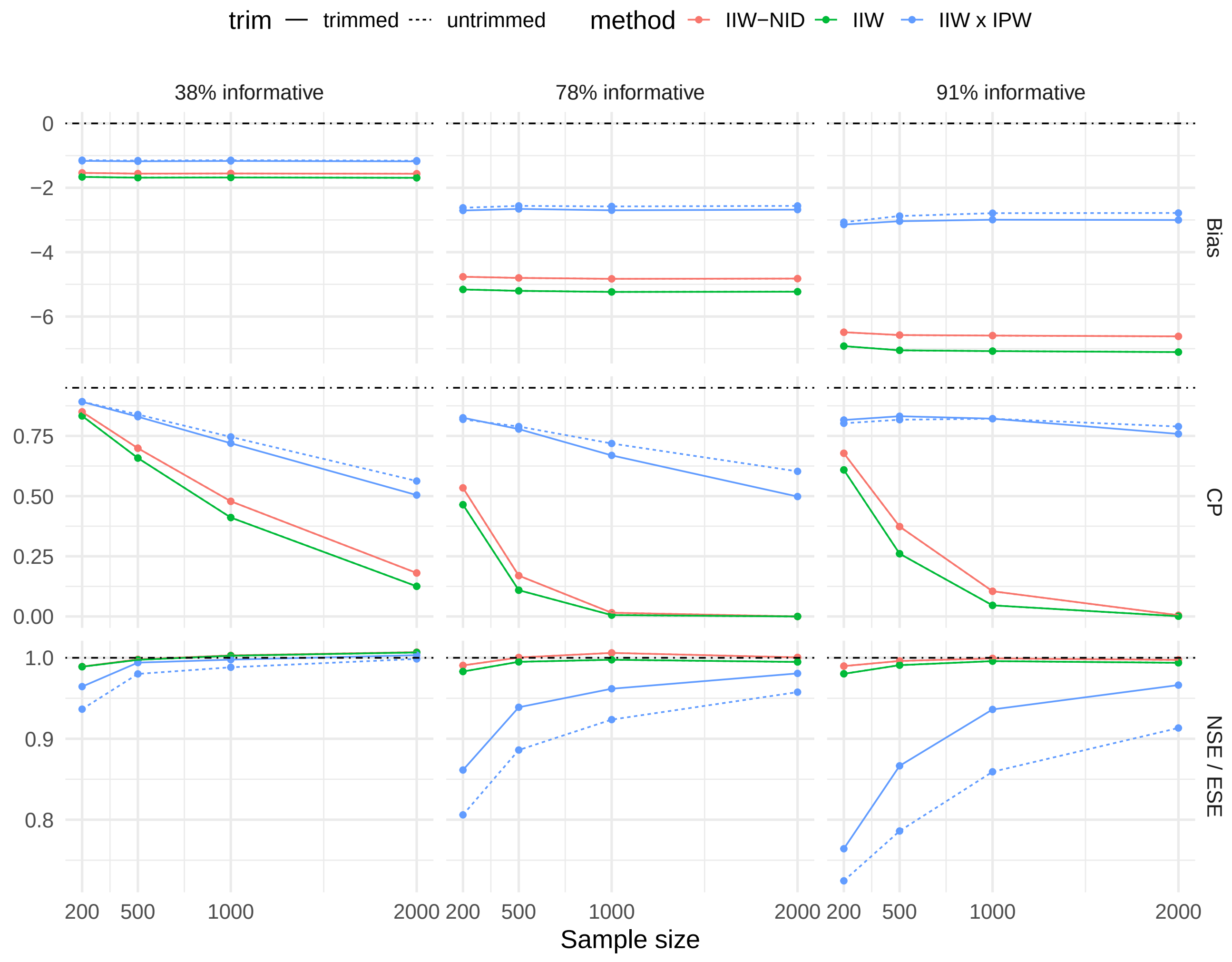

FIGURE A9 Bias, coverage probabilities and the ratio of naive to empirical standard error for AUC in Scenario 1, varying η_0 to achieve different proportions of informative dropout, and misspecifying η_1 as constant, where the true $\eta_1(t) = 0.5 + 0.0625t$. All three methods are compared, with our method in blue. 99.9th percentile-trimmed weights are shown along with untrimmed weights for each case. Dashed lines represent zero bias, 95% coverage probability, and a ratio of 1, respectively.

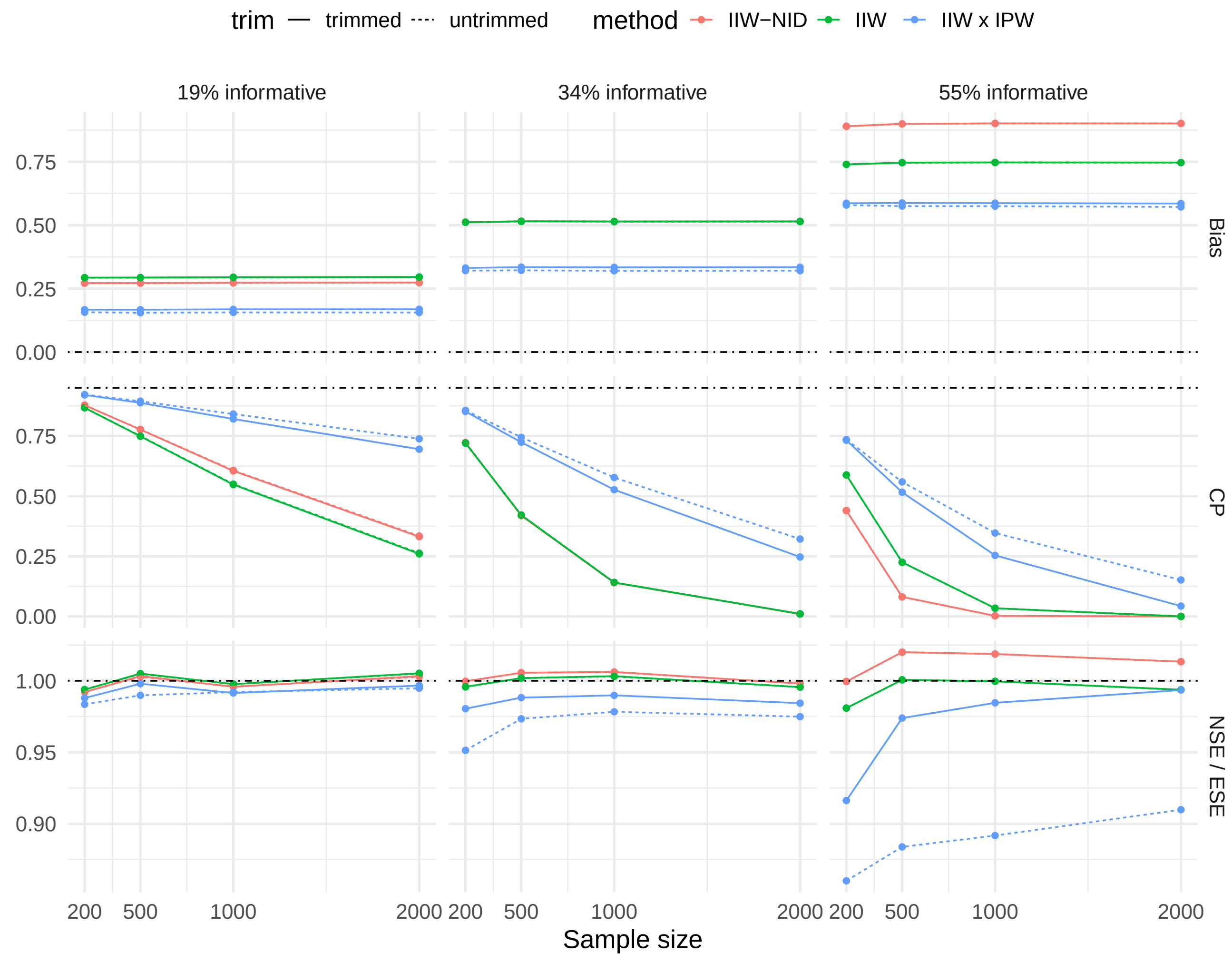

FIGURE A10 Bias, coverage probabilities and the ratio of naive to empirical standard error for AUC in Scenario 2, varying η_0 to achieve different proportions of informative dropout, and misspecifying η_1 as constant, where the true $\eta_1(t) = -0.5 - 0.857t$. All three methods are compared, with our method in blue. 99.9th percentile-trimmed weights are shown along with untrimmed weights for each case. Dashed lines represent zero bias, 95% coverage probability, and a ratio of 1, respectively.

B STAR*D DEMOGRAPHIC VARIABLES

Table B3 summarizes several demographic variables available in the STAR*D dataset which are used in the visit intensity model. For the categorical variables—all except age and education—we present the number and proportion of values in each level. Age and education are measured in decades, and we present the median, minimum and maximum values. Number and proportion of missing values for each variable are also presented.

Characteristic	Number (%)	Missing (%)
**Sex**		0 (0.0%)
Female	2530 (62.6%)	
Male	1509 (37.4%)	
**Age in decades**		2 (0.0%)
Median [Min, Max]	4.05 [1.81, 7.57]	
**Current residence**		4 (0.1%)
Apartment or condominium	1408 (34.9%)	
Detached house/rowhouse or townhouse/mobile home	2505 (62.0%)	
Other	122 (3.0%)	
**Current marital status**		4 (0.1%)
Married	1332 (33.0%)	
Unmarried	2703 (66.9%)	
**Total number of persons in household**		8 (0.2%)
≤ 2	2045 (50.6%)	
> 2	1986 (49.2%)	
**Currently a student**		4 (0.1%)
No	3442 (85.2%)	
Yes	593 (14.7%)	
**Current employment status**		37 (0.9%)
Employed	2291 (56.7%)	
Retired	233 (5.8%)	
Unemployed	1478 (36.6%)	
**Currently do volunteer work**		8 (0.2%)
No	3452 (85.5%)	
Yes	579 (14.3%)	
**On medical or psychiatric leave**		9 (0.2%)
No	3718 (92.1%)	
Yes	312 (7.7%)	
**Has private insurance**		85 (2.1%)
No	1932 (47.8%)	
Yes	2022 (50.1%)	
**Better able to enjoy things**		16 (0.4%)
Agree	3690 (91.3%)	
Disagree	35 (0.8%)	
Neutral	298 (7.4%)	
**Better able to make important decisions**		16 (0.4%)
Agree	3650 (90.4%)	
Disagree	46 (1.1%)	
Neutral	327 (8.1%)	
**Impact of your family and friends**		18 (0.4%)
Difficult	900 (22.3%)	
Helpful	2345 (58.1%)	
Neutral	776 (19.2%)	
**Number of decades in formal education**		12 (0.3%)
Median [Min, Max]	1.30 [0, 2.70]	

TABLE B3 Other current residence category includes rooming house or hotel; retirement complex or senior nursing; healthcare facility or nursing home; and homeless. Not married includes never married; living with someone; separated; divorced; and widowed. Enjoyment was captured via responses to the statement “If I can get the help I need from a doctor, I believe that I will be much better able to enjoy things.” Making decisions was captured via responses to the statement “If I can get the help I need from a doctor, I believe that I will be better able to make important decisions.”

C INTENSITY MODEL OUTPUT

Table C4 summarizes the intensity rate ratios (IRRs), along with 95% confidence intervals, estimated from the visit intensity model. Estimates associated with the demographic variables in Table B3 are presented, along with those associated with QIDS score and its interactions with the visit profile. In this model, we also use a set of four cubic B-spline basis functions to model the difference between the current day and recommended visit day; we depict in Figure C11 the predicted IRRs based on the estimated B-spline coefficients across a range of differences. Figure C12 depicts the estimated IRRs, along with 95% confidence intervals, for the interaction terms between the discretized variables representing how much the patient has deviated from the visit protocol and number of visits missed relative to expectations up to that point.

Predictor	Level or Units	Intensity Rate Ratio (95% CI)
**Sex**	Male	1.017 (0.982, 1.054)
**Age**	Decades	1.033 (1.017, 1.049)
**Residence**	House	0.999 (0.959, 1.039)
Reference: Apartment	Other	0.955 (0.891, 1.023)
**Marital status**	Married	0.936 (0.899, 0.974)
>2 **people in household**		0.931 (0.896, 0.966)
**Student**	No	1.030 (0.979, 1.084)
**Employment**	Retired	0.957 (0.883, 1.036)
Reference: Employed	Unemployed	0.965 (0.928, 1.004)
**Volunteer**	Yes	1.022 (0.975, 1.072)
**Medical/psychiatric leave**	Yes	0.935 (0.875, 0.999)
**Has private insurance**	Yes	1.016 (0.977, 1.056)
**Enjoyment**	Disagree	0.998 (0.808, 1.234)
Reference: Agree	Neutral	1.034 (0.959, 1.115)
**Decision-making**	Disagree	1.010 (0.838, 1.217)
Reference: Agree	Neutral	0.962 (0.895, 1.034)
**Impact of family & friends**	Helpful	1.007 (0.965, 1.05)
Reference: Difficult	Neutral	0.980 (0.929, 1.034)
**Education**	Decades	1.281 (1.209, 1.357)
**QIDS score and visit profile**		
7 days into next visit sequence		2.702 (2.364, 3.088)
1 visit more than expected & early visit		3.955 (2.456, 6.369)
QIDS score at previous visit & ≥ 11 days into next visit sequence		1.042 (1.038, 1.045)
QIDS score at previous visit & < 11 days into next visit sequence		0.920 (0.914, 0.926)

TABLE C4 Intensity rate ratios (along with 95% confidence intervals) associated with the demographic variables as well as those associated with interactions between the QIDS score and the visit profile.

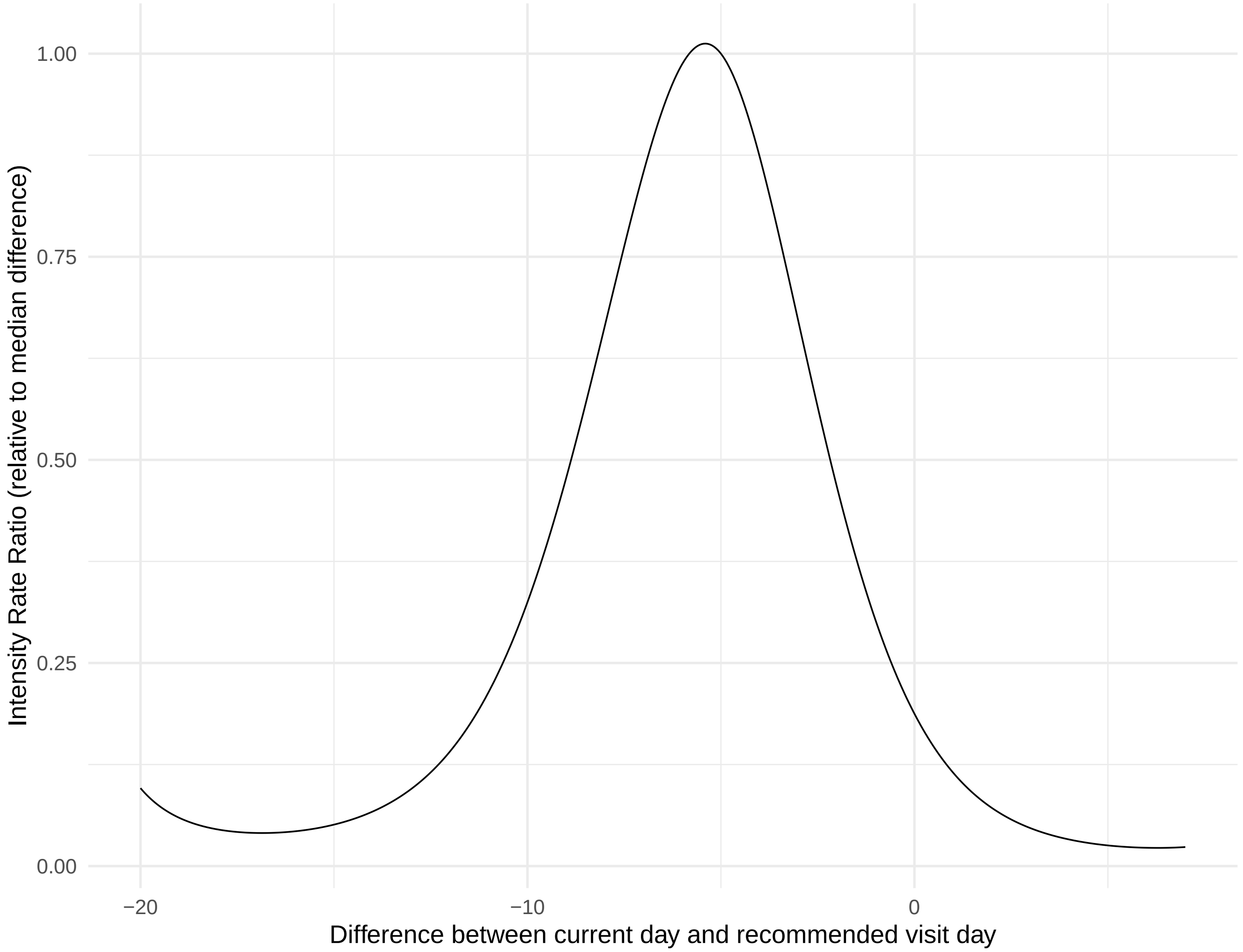

FIGURE C11 Predicted intensity rate ratios across the range of differences between current day and recommended visit day (relative to median difference), based on the estimated B-spline coefficients.

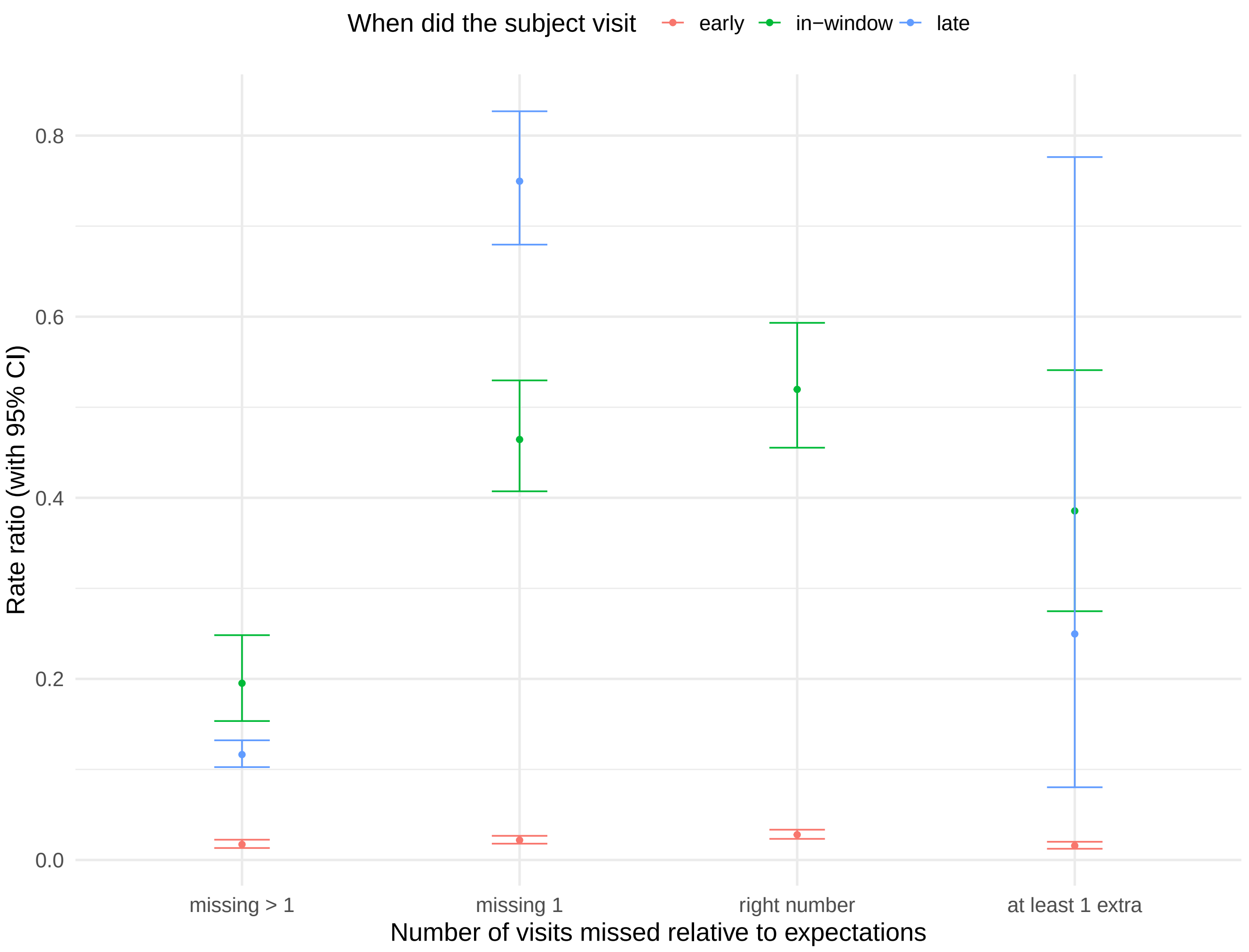

FIGURE C12 Intensity rate ratios (along with 95% confidence intervals) associated with the interaction between the discretized variables representing when the subject visited relative to recommendation and the difference between actual and expected number of visits, respectively.

D DROPOUT MODEL OUTPUT

Figure D13 depicts the odds ratios, along with 95% confidence intervals, estimated from the dropout model. The odds ratios are associated with the interaction terms between the discretized variables for follow-up time and percentage change in QIDS score since baseline.

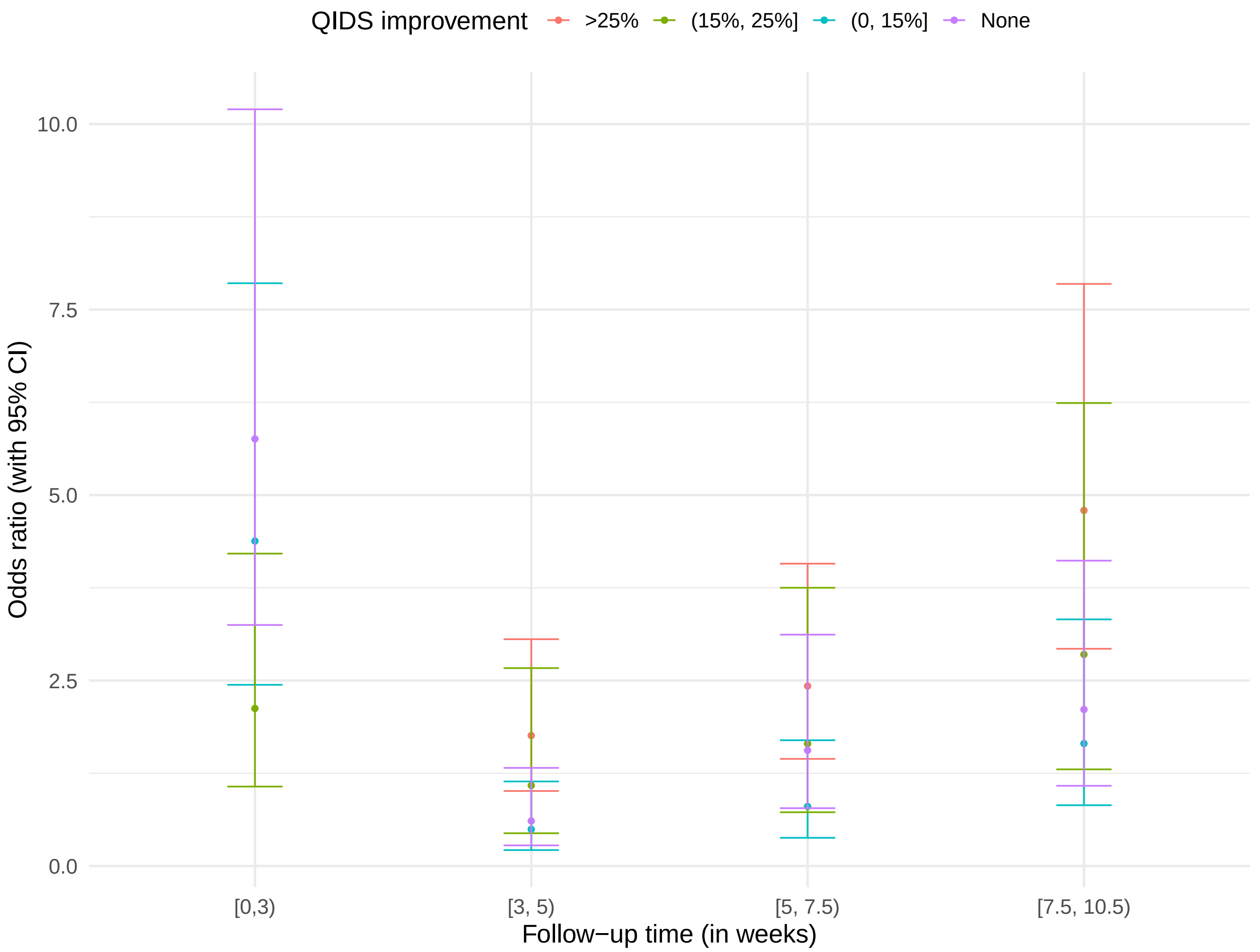

FIGURE D13 Odds ratio estimates (with 95% confidence intervals) from the dropout model for every combination of discretized follow-up time and percent improvement in QIDS score relative to baseline.

E DERIVING THE EXPLICIT FORM OF THE OBSERVED VISIT MODEL

We derive equation (2) using assumptions (3) and (4), as well as the assumption that the competing event time is predictable.

$$\begin{aligned}
\mathbb{E}[\mathrm{d}N_i(t)|\mathcal{H}_i^O(t^-),\boldsymbol{X}_i(t)] &= \mathbb{E}[\mathrm{d}N_i^*(t)\zeta_i(t)|\mathcal{H}_i^O(t^-),\boldsymbol{X}_i(t)] \\
&= \mathbb{E}[\mathrm{d}N_i^*(t)|\mathcal{H}_i^O(t^-),\boldsymbol{X}_i(t)]\mathbb{E}[\zeta_i(t)|\mathcal{H}_i^O(t^-),\boldsymbol{X}_i(t)] \\
&= e^{\gamma_0^\top \mathcal{H}_i^O(t^-)}\mathrm{d}\Lambda_0(t)\mathbb{E}(\mathbb{1}(D_i > t)\mathbb{1}(G_i > t)\mathbb{1}(L_i \geq t)|\mathcal{H}_i^O(t^-),\boldsymbol{X}_i(t)) \\
&= \mathbb{1}(L_i \geq t)e^{\gamma_0^\top \mathcal{H}_i^O(t^-)}\mathrm{d}\Lambda_0(t)\mathbb{E}(\mathbb{1}(D_i > t, G_i > t)|\mathcal{H}_i^O(t^-),\boldsymbol{X}_i(t)) \\
&= \mathbb{1}(L_i \geq t)e^{\gamma_0^\top \mathcal{H}_i^O(t^-)}\mathrm{d}\Lambda_0(t)\mathrm{P}(D_i > t, G_i > t|\mathcal{H}_i^O(t^-),\boldsymbol{X}_i(t)) \\
&= \mathbb{1}(L_i \geq t)e^{\gamma_0^\top \mathcal{H}_i^O(t^-)}\mathrm{d}\Lambda_0(t)\mathrm{P}(D_i > t|G_i > t, \mathcal{H}_i^O(t^-),\boldsymbol{X}_i(t))\mathrm{P}(G_i > t|\mathcal{H}_i^O(t^-),\boldsymbol{X}_i(t))
\end{aligned}$$

F SHOWING THE ESTIMATING EQUATION HAS MEAN ZERO

We use the iterated expectation property to condition on $\boldsymbol{X}_i(t)$:

$$\begin{aligned}
\mathbb{E}[\boldsymbol{U}(\boldsymbol{\beta};\hat{\gamma},\hat{\boldsymbol{\eta}},h)] = \mathbb{E}\Bigg\{&\sum_{i=1}^{n}\int_0^\infty \boldsymbol{X}_i(t)\left\{\left.\frac{\mathrm{d}g(\mu)}{\mathrm{d}\mu}\right|_{\mu_i(t;\boldsymbol{\beta})}\right\}^{-1} v(\mu_i(t;\boldsymbol{\beta}))^{-1}h(\boldsymbol{X}_i(t)) \\
&\times \mathbb{E}\left[\{Y_i(t)-\mu_i(t;\boldsymbol{\beta})\}\frac{\mathrm{d}N_i(t)}{e^{\gamma_0^\top \mathcal{H}_i^O(t^-)}\mathrm{P}(D_i > t|G_i > t, \mathcal{H}_i^O(t^-),\boldsymbol{X}_i(t))}\right|\boldsymbol{X}_i(t)\Bigg]\Bigg\}
\end{aligned}$$

It remains to show that the expected value of the term in the square parentheses above is equal to zero, given our assumptions.

$$\begin{aligned}
&\mathbb{E}\left[\{Y_i(t)-\mu_i(t;\boldsymbol{\beta})\}\frac{\mathrm{d}N_i(t)}{e^{\gamma_0^\top \mathcal{H}_i^O(t^-)}\mathrm{P}(D_i > t|G_i > t, \mathcal{H}_i^O(t^-),\boldsymbol{X}_i(t))}\middle|\boldsymbol{X}_i(t)\right] \\
&= \mathbb{E}\left[\{Y_i(t)-\mu_i(t;\boldsymbol{\beta})\}\frac{\mathbb{E}[\mathrm{d}N_i(t)|\mathcal{H}_i^O(t^-),\boldsymbol{X}_i(t),Y_i(t)]}{e^{\gamma_0^\top \mathcal{H}_i^O(t^-)}\mathrm{P}(D_i > t|G_i > t, \mathcal{H}_i^O(t^-),\boldsymbol{X}_i(t))}\middle|\boldsymbol{X}_i(t)\right] \\
&= \mathbb{E}\left[\{Y_i(t)-\mu_i(t;\boldsymbol{\beta})\}\frac{\mathbb{E}[\mathrm{d}N_i(t)|\mathcal{H}_i^O(t^-),\boldsymbol{X}_i(t)]}{e^{\gamma_0^\top \mathcal{H}_i^O(t^-)}\mathrm{P}(D_i > t|G_i > t, \mathcal{H}_i^O(t^-),\boldsymbol{X}_i(t))}\middle|\boldsymbol{X}_i(t)\right] \\
&= \mathbb{E}\left[\{Y_i(t)-\mu_i(t;\boldsymbol{\beta})\}\mathbb{1}(L_i \geq t)\frac{e^{\gamma_0^\top \mathcal{H}_i^O(t^-)}\mathrm{d}\Lambda_0(t)\mathrm{P}(D_i > t|G_i > t, \mathcal{H}_i^O(t^-),\boldsymbol{X}_i(t))\mathrm{P}(G_i > t|\mathcal{H}_i^O(t^-),\boldsymbol{X}_i(t))}{e^{\gamma_0^\top \mathcal{H}_i^O(t^-)}\mathrm{P}(D_i > t|G_i > t, \mathcal{H}_i^O(t^-),\boldsymbol{X}_i(t))}\middle|\boldsymbol{X}_i(t)\right] \\
&= \mathbb{E}\left[\{Y_i(t)-\mu_i(t;\boldsymbol{\beta})\}\,\mathrm{d}\Lambda_0(t)\mathbb{1}(L_i \geq t)\mathrm{P}(G_i > t|\mathcal{H}_i^O(t^-),\boldsymbol{X}_i(t))\middle|\boldsymbol{X}_i(t)\right] \\
&= \mathbb{E}\left[\{Y_i(t)-\mu_i(t;\boldsymbol{\beta})\}\,\mathrm{d}\Lambda_0(t)\mathbb{1}(L_i \geq t)\mathbb{E}(\mathbb{1}(G_i > t)|\mathcal{H}_i^O(t^-),\boldsymbol{X}_i(t))\middle|\boldsymbol{X}_i(t)\right] \\
&= \mathbb{E}\left[\{Y_i(t)-\mu_i(t;\boldsymbol{\beta})\}\,\mathrm{d}\Lambda_0(t)\mathbb{1}(L_i \geq t)\mathbb{1}(G_i > t)\middle|\boldsymbol{X}_i(t)\right] \\
&= \mathrm{d}\Lambda_0(t)\mathbb{E}[\mathbb{1}(G_i > t)|\boldsymbol{X}_i(t)]\mathbb{E}\left[\mathbb{E}\left[\{Y_i(t)-\mu_i(t;\boldsymbol{\beta})\}\,\mathbb{1}(L_i \geq t)\middle|\boldsymbol{X}_i(t),\xi_i^L(t)=0\right]\middle|\boldsymbol{X}_i(t)\right] \\
&= \mathrm{d}\Lambda_0(t)\mathrm{P}(G_i > t|\boldsymbol{X}_i(t))\mathbb{E}\left[\mathbb{1}(L_i \geq t)\mathbb{E}\left[\{Y_i(t)-\mu_i(t;\boldsymbol{\beta})\}\middle|\boldsymbol{X}_i(t),\xi_i^L(t)=0\right]\middle|\boldsymbol{X}_i(t)\right] = 0
\end{aligned}$$

since the innermost expectation is equal to zero by assumption.
